# Ferrites: magnetic materials as an alternate source of green electrical energy

**DOI:** 10.1016/j.heliyon.2019.e01151

**Published:** 2019-01-24

**Authors:** Pranati Kharbanda, Tushar Madaan, Isha Sharma, Shruti Vashishtha, Parveen Kumar, Arti Chauhan, Sumit Mittal, Jarnail S. Bangruwa, Vivek Verma

**Affiliations:** a Department of Physics, Hindu College, University of Delhi, Delhi, India; b Department of Physics and Astrophysics, University of Delhi, Delhi, India

**Keywords:** Materials science

## Abstract

Ferrites samples Mg_1-x_Li_x_Fe_2_O_4_ for x = 0.0, 0.1, 0.2, 0.3, were synthesized by solid-state sintering method. Detailed investigations were made on the structural, morphological, magnetic and electrical proprieties of these samples. A detailed investigation was performed on power generation of these samples and role of Li-doping has been discussed. The X-ray Diffraction (XRD) pattern confirms the spinel phase formation in samples without any impurity. It is observed from Scanning Electron Microscopy that average grain size of samples decreases with lithium doping in MgFe_2_O_4_. The saturation magnetization of MgFe_2_O_4_ (15.4 emu/g) is found to increase with Lithium percentage and maximum 39.3 emu/g for Mg_0.7_Li_0.3_Fe_2_O_4_ sample. Ferrites play a crucial role in magnetic recording, microwave magnetic devices and many applications in medical sciences. Recently, it was observed that ferrites can be an alternate source of green energy by inventing hydroelectric cell (HEC). The processes of water adsorption and dissociation on the metal-oxide surface, plays an important role in production of electricity in ferrites. When, water is sprayed on hydroelectric cell the thermodynamic driving force is responsible for the formation of stable metal-oxygen or metal-hydroxyl bonds. The reactivity of ferrite surface towards water is based on the interaction of these ions and the d orbital of the Fe atom. Due to this interaction, water dissociated in H_3_O^+^ and OH^−^ ions and migrates toward silver and zinc electrodes respectively. A typical hydroelectric cell of 2 inch diameter produces 17.1 mA of peak current and 949 mV voltage with a maximum output power of 15.85 mW for Li = 0.2 doped MgFe_2_O_4_ sample.

## 1 Introduction

Ferrites belong to a group of magnetic materials extensively used in many applications such as microwave devices, computer memory chip, magnetic recording media, radio frequency coil fabrication, transformer cores, rod antennas and many branches of telecommunication and electronic engineering [1, 2, 3, 4, 5]. It is due to its attractive features such as high Curie temperature, high saturation magnetization and square hysteresis loop. Most of the soft ferrites have spinel structure which consists of the 8 formula units [6, 7] of M^2+^Fe_2_^3+^O_4_. Normally ‘M’ is a divalent atom of radius between 0.80 Å to 1.0 Å, such as Mn, Mg, Cd, Fe, Zn, Cu etc and Fe_2_^3+^ is a trivalent atom. The structure of spinel ferrite is complex, which consists of a cubic closed-packed array of 32 oxide ions, which forms 64 tetrahedral and 32 octahedral site in one unit cell [containing eight formula units (M^2+^Fe_2_^3+^O_4_)_8_]. These unit cells contain two kinds of sites. One is called octahedral or B-site, in which the oxygen ions around it occupy the corner of an octahedron. The other is called as tetrahedral or A site, in which M^2+^ is located at the centre of a tetrahedron whose corners are occupied by oxygen ions [8]. The cation distribution on A and B sites, in general is represented by: [M^2+^_δ_ Fe^3+^_1-δ_ ]_A_ [M^2+^_1-δ_ Fe^3+^_1+δ_]_B_ O^2-^_4_, where δ = 1 for normal spinel structure and δ = 0 for inverse spinel structure. The distribution of cations among the tetrahedral and octahedral site play an important role in deciding the electric, dielectric, magnetic and structural properties of spinel ferrites [9, 10].

**Fig. 1:**
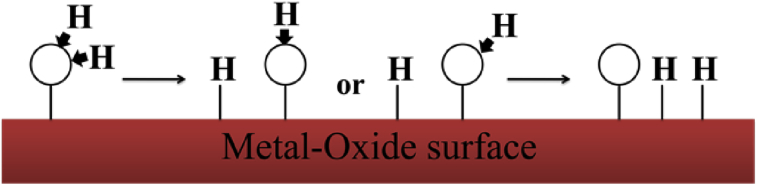
Schematic representation of possible dissociation of adsorbed water.

Metal-oxide surfaces offer great potential for a wide variety of applications like magnetic recording media, water-gas shift reaction, photocatalytic splitting etc. [11, 12, 13]. Generation of electric current and voltage by a hydroelectric cell using dissociation of water has unfolded new green electrical energy source [14]. The adsorption of water on oxide surface can be in different manner. First, well-ordered ionic single-crystal samples are often nonreactive for H_2_O dissociation. It may be due to that the dissociation of water on the surface is by reaction at the defect sites and facet edges in the oxide polycrystalline samples, and defect sites are very less on the single crystals [15]. The influence of defect lattice sites and oxygen deficit surfaces are important for dissociation of water. The second distinctive feature of H_2_O adsorption is the formation of relatively strong chemisorption bonds on some ionic surfaces, so that molecular adsorbed H_2_O can be stable even at room temperature [16]. Water adsorption at metal-oxide surfaces is governed by a subtle balance between water-water hydrogen bonding and water-metal oxide interactions, which together determine the stability of the water structures formed. Adsorbed water can dissociate in hydroxyl, atomic oxygen and atomic hydrogen as experimentally observed by many researchers [17, 18]. The possible dissociation pathways of water are illustrated schematically in Fig. 1.

In this work, we have reported the effect of Li doping on the structural, magnetic and electrical properties of magnesium ferrite. A detailed investigation has been made on electric current and voltage generated by a series of samples of Mg_1-x_Li_x_Fe_2_O_4_.

## 2 Experimental

**Fig. 2:**
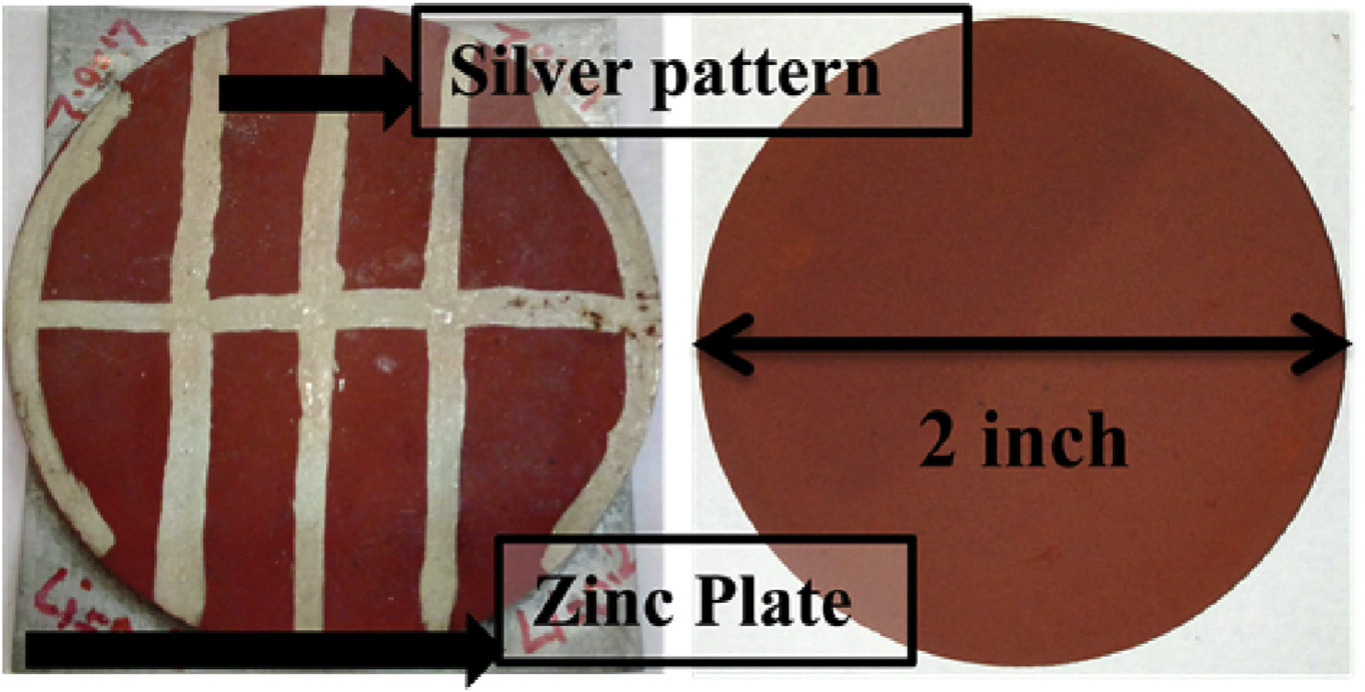
Image of ferrites pellets with silver comb pattern and zinc plate work as hydroelectric cell.

The samples were prepared by the conventional double sintering ceramic technique, in which oxides or carbonates were used as precursors. According to the stoichiometric composition of Mg_1-x_Li_x_Fe_2_O_4_ (x = 0.0, 0.1, 0.2, 0.3), specified molar ratio of the magnesium Oxide (MgO), iron oxide (Fe_2_O_3_) and lithium carbonate (Li_2_CO_3_) were mixed and ground by wet grinding in distilled water. Mixed powders were pre-sintered at 750 °C for 5 h in ambient atmosphere. After pre-sintering the obtained powders were again mixed and grinded followed by pellets formation. Two kinds of pellets were prepared for each sample, one was two inch of diameter for hydroelectric cell and second was 8 mm in diameter for electrical characterization. These obtained pellets of all samples were finally sintered at 1050 °C for 5 h. Heating and cooling rate was controlled at 5 °C/min. Finally, obtained pellets of 8 mm diameter were silver coated on the opposite faces for electrical characterization. And in pellet of two inch diameter a zinc plate was fix on the one face and other face was coated with silver in comb pattern for hydroelectric characterization as shown in Fig. 2. The structural characterization of samples was carried out by the X-ray diffraction (XRD Rigaku Miniflex II, step size = 0.02°) technique using Cu Kα radiation (wavelength λ = 1.5406 Å). A scanning electron microscope (JEOL/JSM-6610LV) was used to observe the microstructure details of the samples. Magnetic measurements were performed using vibrating sample magnetometer (VSM, Lake Shore7304) for all samples at room temperature. Current and voltage generated by each hydroelectric cell was measured by digital multi meter (DMM). A.C. resistivity of samples was measured by using Novocontrol technologies Alpha-A high performance Frequency Analyzer.

## 3 Results and discussion

### 3.1 Structural analysis

**Fig. 3:**
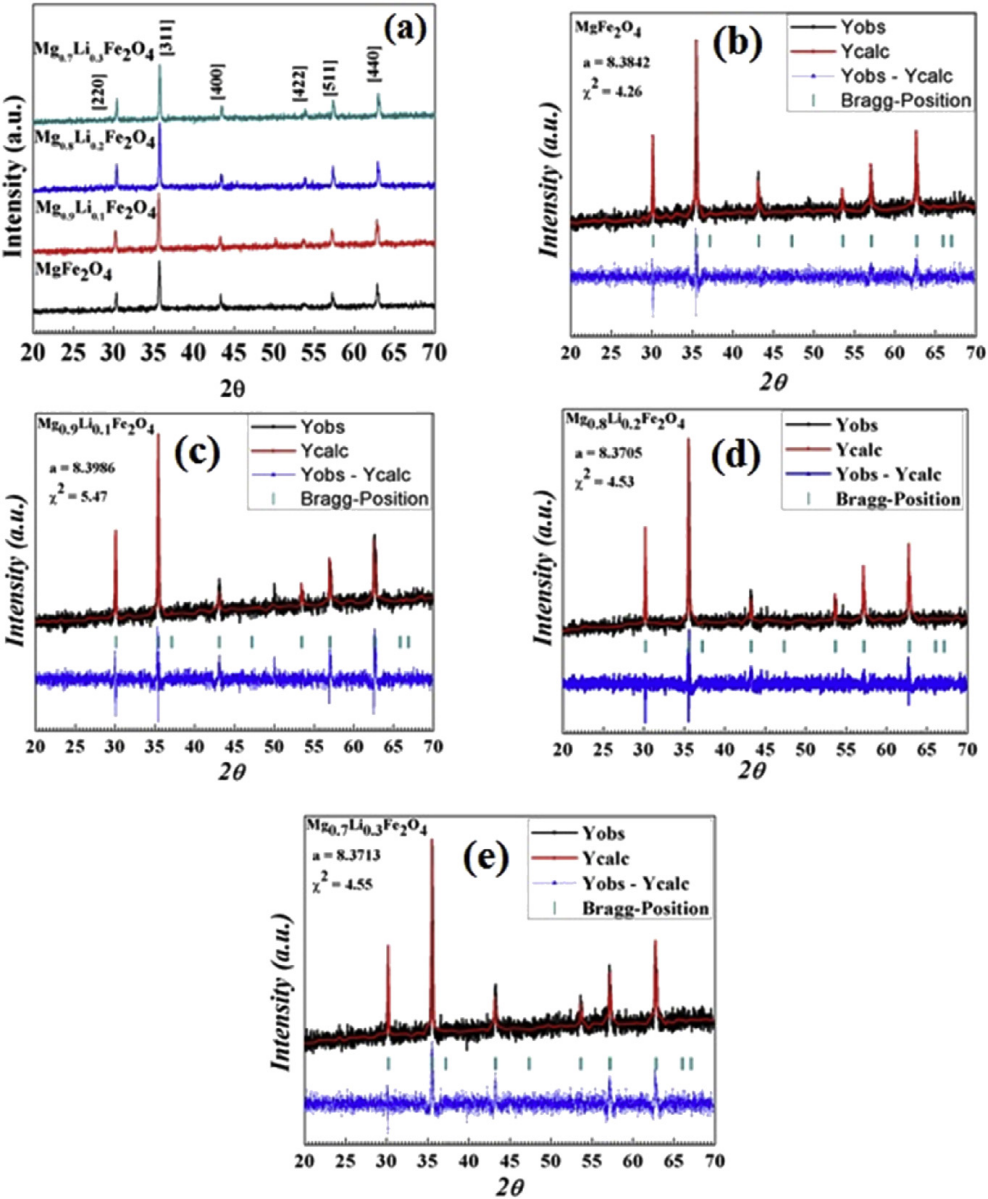
(a) X-ray powder diffraction pattern of Mg_1-x_Li_x_Fe_2_O_4_ samples and Rietveld Refinement patterns of (b) x = 0.0, (c) x = 0.1, (d) x = 0.2, (e) x = 0.3 samples.

Fig. 3 shows the X-ray powder diffraction pattern of Mg_1-x_Li_x_Fe_2_O_4_ (0 ≤ x ≤ 0.3) samples. This can be observed from the XRD pattern of MgFe_2_O_4_, the obtained peak positions are same as reported in JCPDS file (73-1720), which exhibits the inverse-spinel phase formation with Fd$\bar{3}$m space group. FullProf programme have been used for Rietveld refinement technique to analyze the XRD patterns of samples. The structural model Fd$\bar{3}$m (space group) and initial structural parameters (Wyckoff positions) of metal 8a, iron 16d and oxygen atoms 32e have been taken for all samples. The method employs a least-squares procedure to compare Bragg's intensities and those calculated from a possible structural model. The fitting quality of the experimental data is assessed by computing the parameters such as ‘goodness of fit χ^2^ and R factors [19]. The typical Rietveld refinement X-ray pattern for all samples is given in Fig. 3. Fitting parameters, goodness of fit (χ^2^) and lattice constant and X-ray density calculated by the Rietveld analysis are listed in Table 1. The lattice parameter value increases from 8.3842 Å to 8.3713 Å which indicates the incorporation of Li^+^ ions in the Magnesium ferrite.

**Table 1:** Structural parameters of Mg_1-x_Li_x_Fe_2_O_4_ samples.

Samples	Lattice parameter a = b = c (Ǻ)	χ^2^	X-ray density (g/cc)
MgFe_2_O_4_	8.3842	4.26	4.51
Mg_0.9_Li_0.1_Fe_2_O_4_	8.3986	5.47	4.45
Mg_0.8_Li_0.2_Fe_2_O_4_	8.3705	4.53	4.44
Mg_0.7_Li_0.3_Fe_2_O_4_	8.3713	4.55	4.41

### 3.2 Scanning Electron Microscopy (SEM)

**Fig. 4:**
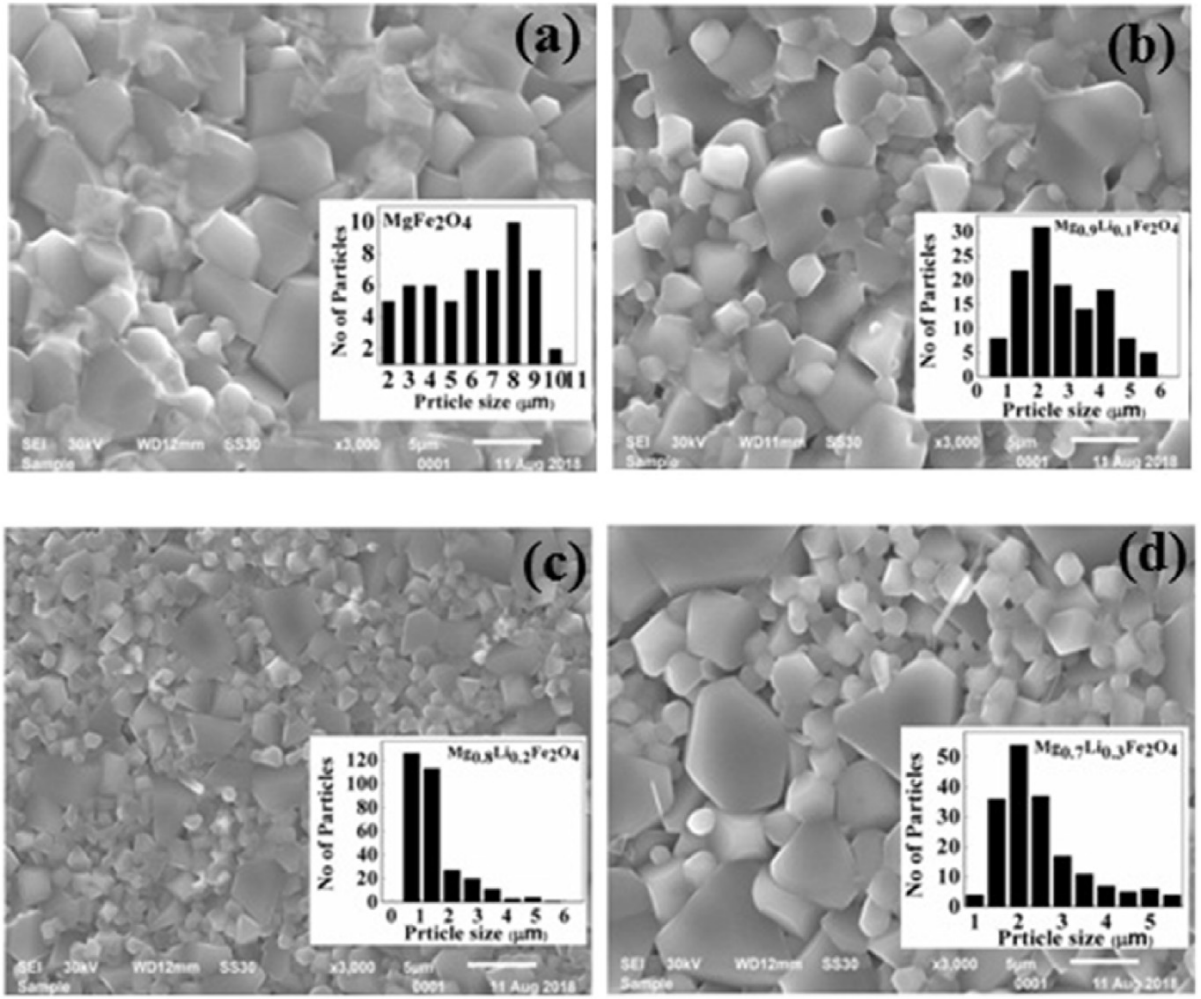
SEM images of Mg_1-x_Li_x_Fe_2_O_4_ samples (a) x = 0.0, (b) x = 0.1, (c) x = 0.2, (d) x = 0.3 and their grain size distribution (in-set).

Magnetic and electrical properties of ferrite samples are strongly dependent on their microstructure. Typical SEM images of the sintered pellets for the Mg_1-x_Li_x_Fe_2_O_4_ (0 ≤ x ≤ 0.3) samples are shown in Fig. 4. It can be observed that the micrographs of all samples exhibit randomly oriented fine crystalline structure. The grains of Mg_1-x_Li_x_Fe_2_O_4_ samples are found to be closely packed. All samples are mostly homogeneous in nature but with different grain size distribution, which may be due to the difference in growth rate of individual phases in the samples.

We can observe that the pure sample MgFe_2_O_4_ exhibits dual average sizes, 3–4 μm and 8μm of grain distribution. We can observe that densification increases as we increase the amount of lithium in MgFe_2_O_4_. The dual nature and grain size growth is decreasing with lithium doping. Mg_0.8_Li_0.2_Fe_2_O_4_ sample shows highly dense microstructure with average grain size 1–2 μm. Reduction in grain size due to doping may arise due to different diffusion rates of constituting elements of the compounds [20]. This modified microstructure may enhance the magnetic and electrical properties of ferrite samples.

### 3.3 Magnetic properties

**Fig. 5:**
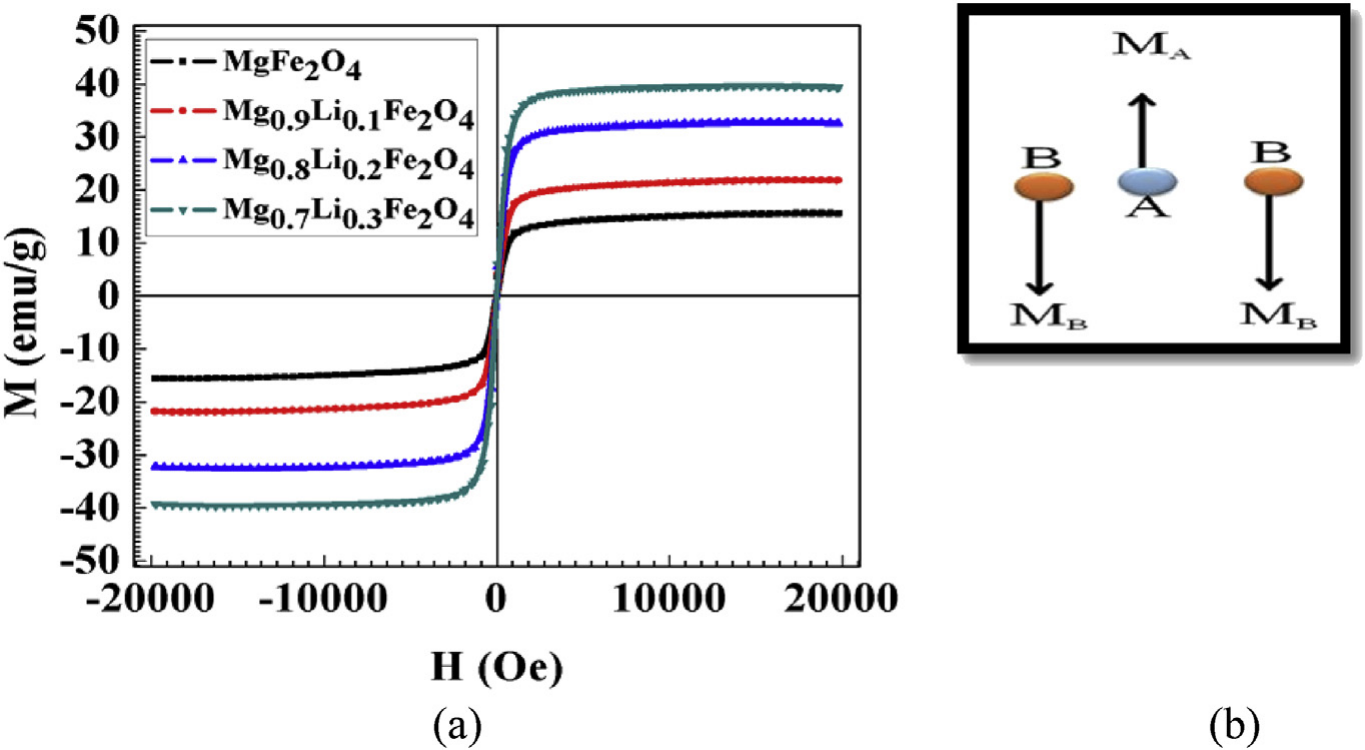
(a) M-H loops of Mg_1-x_Li_x_Fe_2_O_4_ samples at room temperature and (b) Neel's collinear model to explain the magnetic behaviour in ferrites.

The M-H loops for all samples have been recorded from VSM up to the maximum field of ± 2T at room temperature, shown in Fig. 5. All samples are showing well saturated M-H loop. It is observed that the saturation magnetization increases slowly with increase in Li concentration in Mg_1-x_Li_x_Fe_2_O_4_ ferrites as shown in Table 2. It is well known that magnesium ferrite is inverse spinel with cations distribution as [${\text{Mg}}_{1-\text{x}}^{2+}{\;\text{Fe}}_{\text{x}}^{3+}]$_A_ [${\text{Mg}}_{\text{x}}^{2+}{\;\text{Fe}}_{2-\text{x}}^{3+}]$_B_ O_4_, where x represents the degree of inversions, which is defined as the fraction of the (A) sites occupied by Fe^3+^ cations [21]. The magnetic properties in the ferrites depend on the interaction between the magnetic moments on the tetrahedral (A-site) and octahedral (B-site) sites and their exchange interactions. In a spinel ferrite, each ion at A-site has 12 B-site ions as nearest neighbours while a B-site ion has six A-site and six B-site ions nearest neighbours. According to Neel's two sublattice model, the A-B super exchange interaction pre-dominant to the sublattice A-A and B-B interactions. A pictorial presentation of alignment of A and B-site magnetic moments according to Neel's model is given in Fig. 5(b). Hence the net magnetization of spinel ferrites is given by **μ = μ**_**B**_ **- μ**_**A**_ [22]. Lithium doping in the MgFe_2_O_4_ may reduce the A-B interaction which increases the magnetic moment in the compounds. An increase in saturation magnetization is observed with Li contain which may be due to replacement of Mg^2+^ ions with Li^+^ in A-site and simultaneous migration of Fe^3+^ ions from A-sites to B-sites as reported by S. Rahman et al. [23].

**Table 2:** Observed magnetic parameters from M-H loops of Mg_1-x_Li_x_Fe_2_O_4_ samples at room temperature.

Sample	Hc (Oe)	Mr (emu/g)	Ms (emu/g)
MgFe_2_O_4_	96.4	1.9	15.4
Mg_0.9_Li_0.1_Fe_2_O_4_	81.1	2.4	21.7
Mg_0.8_Li_0.2_Fe_2_O_4_	61.9	2.9	32.6
Mg_0.7_Li_0.3_Fe_2_O_4_	52.3	3.3	39.3

### 3.4 Hydroelectric properties

**Fig. 6:**
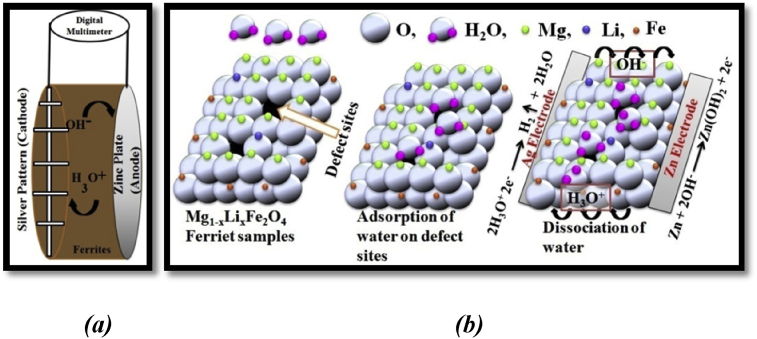
(a) Schematic diagram of working of a hydroelectric cell and (b) representation of water adsorption and its dissociation on the surface of the Mg_1-x_Li_x_Fe_2_O_4_ samples.

The working of a hydroelectric cell is based on adsorption of water molecule and its dissociation and conduction of OH^−^, H_3_O^+^ ions in the ferrites medium. The mechanism of water molecule adsorption and dissociation on ferrites surface is schematically represented in Fig. 6. As the water molecules come in contact with the ferrite surface, immediately water molecules dissociate into hydronium (H_3_O^+^) and hydroxide (OH^−^) ions [14]. Hyronium ions are reduced at Ag electrode to release hydrogen gas and hydroxide ions are oxidized at Zn electrode as shown by Eqs. i, ii, iii).

(i) 4H_2_O → 2H_3_O^+^ + 2OH^−^ (dissociation of water molecules)

(ii) Zn + 2OH^−^ → Zn(OH)_2_ + 2e^−^ E_oxd_ = −0.76 V (at Zn electrode)

(iii) 2e^−^ + 2H_3_O+ → 2H_2_O + H_2_ E_red_ = 0.22 V (at silver electrode)

As we can observe that the overall voltage generated between the silver and Zn electrodes is E_cell_ = 0.22 + 0.76 = 0.98 V. This voltage produces an electric field between the electrodes which helps in further motion of ions (H_3_O^+^, OH^−^) in the ferrite medium.

**Fig. 7:**
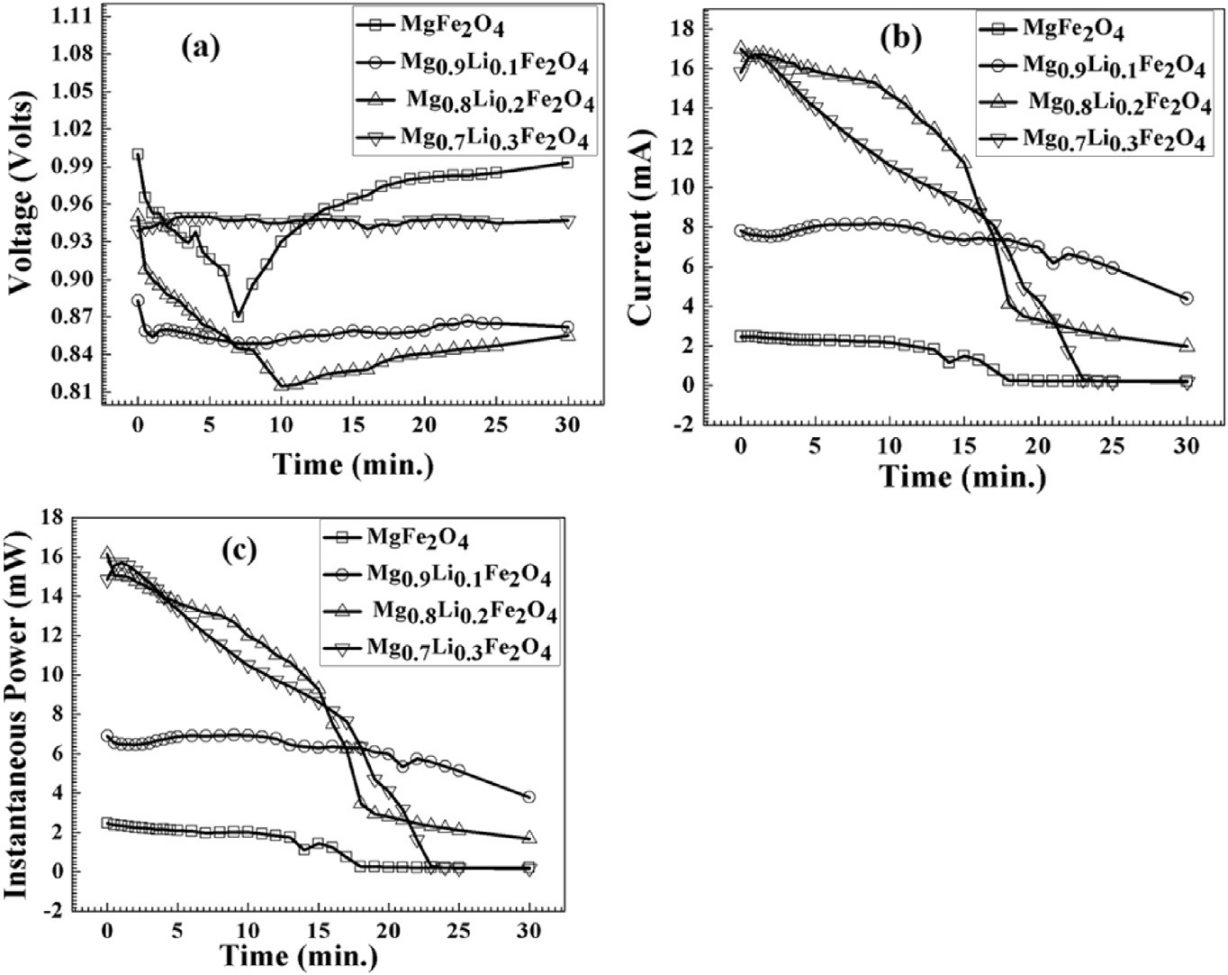
(a) Voltage, (b) current and (c) power generated by hydroelectric cells of the Mg_1-x_Li_x_Fe_2_O_4_ samples.

Dissociation of water molecule occurs on the surface of Mg_1-x_Li_x_Fe_2_O_4_ samples due to attraction between oxygen lone pair electron of H_2_O molecule and Fe^3+^ ions situated at octahedral site, which make strong chemisorption bond in between [24]. Oxygen defects also have been observed play an important role in dissociation of water molecule [25]. Hydroelectric cells of diameter 2 inch fabricated in lab as shown in Fig. 2. We recorded the open circuit voltage and short circuit current for each samples for 30 minutes after dipping in deionized water to analyze the hydroelectric properties of Mg_1-x_Li_x_Fe_2_O_4_ cells. We repeated such observations in sets of samples to observe the consistency of results. All the observations have been performed at room temperature and in ambient atmosphere. These hydroelectric cells produced the emf in the range of 859–965 mV and maximum current in the range of 17–2.47 mA as shown in Fig. 7. We observed some fluctuation in open voltage at initial stage (5–10 min.) which becomes almost constant which sustained for more than 24 hours. But the response of current is not up to the values reported by R. K. Kotnala et al. [14]. We observed that current decreases very fast with time. This decrease in current could be a result of saturation of Fe-sites in octahedral due to involvement in transportation of OH^−^ and H_3_O^−^ ions in the ferrite medium. The decrease in the current of hydroelectric cells is the main barrier for large energy production for longer time. Further investigation is required to rectify this problem. We observed that Li doping improved the voltage and current (power) response in the ferrite samples as shown in Fig. 7. Maximum current is exhibited by Mg_0.8_Li_0.2_Fe_2_O_4_ sample. Monovalent lithium ions substitute the divalent Magnesium ions which create oxygen vacancy due to charge unbalance in the compounds. This increase in oxygen vacancies with lithium doping may increase chemidissociation which modifies the current in result. But as we discussed, further investigation is required to modify the current in hydroelectric cells for large scale power production.

### 3.5 AC resistivity response

**Fig. 8:**
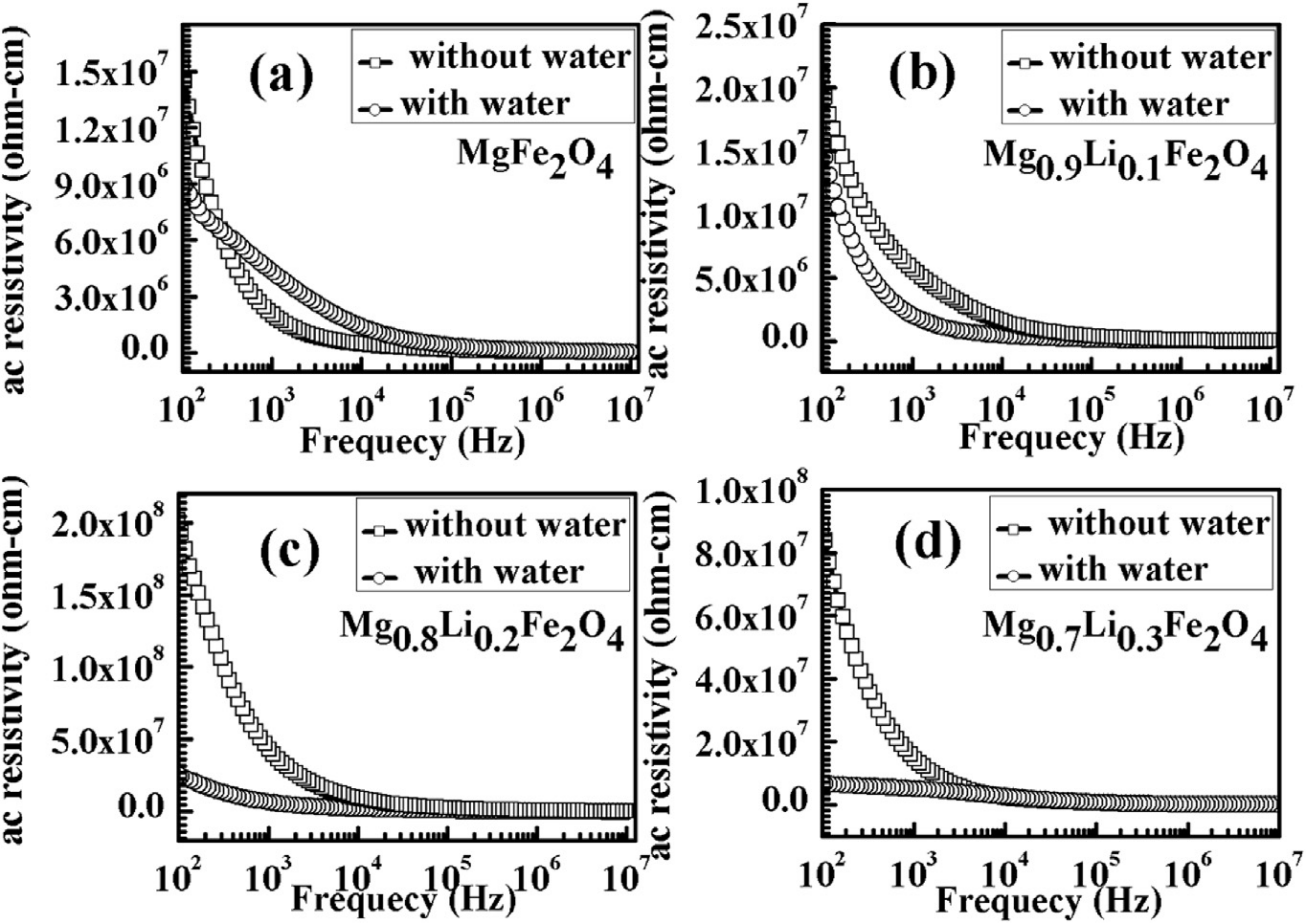
Variation of resistivity of the Mg_1-x_Li_x_Fe_2_O_4_ samples, (a) x = 0.0, (b) x = 0.1, (c) x = 0.2, (d) x = 0.3, with and without water.

Fig. 8 shows the variation of a.c. resistivity of Mg_1-x_Li_x_Fe_2_O_4_ samples with log of frequency at room temperature. Here, we recorded the resistivity of the all dry samples (without water) from 100 Hz to 10 MHz. Then, all samples were dipped in deionized water for some time followed by ac resistivity measurement (with water). From the Fig. 8, we can observe that a.c. resistivity of the samples decrease with the frequency and become almost constant after 10 MHz. This change in the conduction mechanism in the ferrites can be explained on the basis of hopping of charge carriers between Fe^3+^ and Fe^2+^ ions on the octahedral sites [25]. At the higher applied frequency up to 10 MHz the hopping phenomenon increases which resulting in a decrease in the resistivity of the ferrite samples. Further increase in the frequency beyond the 10 MHz the resistivity becomes negligible small and remains constant because the hopping frequency no longer follows the applied field [26]. Resistivity of the Mg_1-x_Li_x_Fe_2_O_4_ samples decreases after dipping into deionized water (resistivity of deionized water in MΩ). The value of resistivity is minimum for Mg_0.8_Li_0.2_Fe_2_O_4_ and Mg_0.7_Li_0.3_Fe_2_O_4_ samples as predicted from Fig. 8. This implies that formation of OH^−^ and H_3_O^+^ ions is more in these samples which may help in conduction. These results are also supported by current generation in hydroelectric cells. The maximum current was generated by the Mg_0.8_Li_0.2_Fe_2_O_4_ and Mg_0.7_Li_0.3_Fe_2_O_4_ samples. These results, confirm that ferrite Mg_1-x_Li_x_Fe_2_O_4_ materials are responsible to dissociate the water molecules in the ions.

## 4 Conclusions

Polycrystalline Mg_1-x_Li_x_Fe_2_O_4_ (0.0 ≤ x ≤ 0.3) ferrite samples have been synthesized by solid state method. Phase purity has been confirmed by XRD analysis. The lattice parameters are found to decrease with Li substitution which was calculated by using Rietveld refinement technique. Surface morphology was observed by using SEM images and shows a decrease in grain size with Li substitution. An increase is also observed in saturation magnetization (M_s_) with Li substitution in Mg_1-x_Li_x_Fe_2_O_4_ samples, which is explained on the basis of Neel's two sub-lattices Model. In hydroelectric cells, voltage, current and power generation has been investigated in detail. Maximum power was observed in Mg_0.8_Li_0.2_Fe_2_O_4_ sample. Resistivity response with frequency of samples without water and with water confirms that ferrite Mg_1-x_Li_x_Fe_2_O_4_ materials are responsible to dissociate the water molecules in ions. But further investigation is required to modify the current in hydroelectric cells for large scale power production. Hence, Ferrites: Magnetic materials may be an alternate source of green and safe electrical energy.

## Declarations

### Author contribution statement

Pranati Kharbanda, Tushar Madaan: Conceived and designed the experiments; Performed the experiments; Contributed reagents, materials, analysis tools or data.

Isha Sharma, Shruti Vashishtha, Arti Chauhan, Sumit Mittal: Performed the experiments; Contributed reagents, materials, analysis tools or data.

Jarnail S. Bangruwa: Analyzed and interpreted the data; Wrote the paper.

Parveen Kumar: Conceived and designed the experiments; Analyzed and interpreted the data; Contributed reagents, materials, analysis tools or data; Wrote the paper.

Vivek Verma: Conceived and designed the experiments; Analyzed and interpreted the data; Wrote the paper.

### Funding statement

This research did not receive any specific grant from funding agencies in the public, commercial, or not-for-profit sectors.

### Competing interest statement

The authors declare no conflict of interest.

### Additional information

No additional information is available for this paper.
